# Additively Manufactured Al/SiC Cylindrical Structures by Laser Metal Deposition

**DOI:** 10.3390/ma13153331

**Published:** 2020-07-27

**Authors:** Ainhoa Riquelme, Pilar Rodrigo, María Dolores Escalera-Rodríguez, Joaquín Rams

**Affiliations:** Escuela Superior de Ciencias Experimentales y Tecnología, Ciencia e Ingeniería de Materiales, Universidad Rey Juan Carlos, 28933 Móstoles, Spain; pilar.rodrigo@urjc.es (P.R.); dolores.escalera@urjc.es (M.D.E.-R.); joaquin.rams@urjc.es (J.R.)

**Keywords:** laser metal deposition, aluminum matrix composites, SiC particles, titanium, additive manufacturing

## Abstract

Preliminary characterization of the microstructure of Al/SiCp composites prepared by Laser Metal Deposition (LMD) was analyzed, and the microhardness and wear behavior of the materials manufactured have been evaluated. It has been determined that the combined effect of the laser speed and power is decisive for the fabrication process. The microstructure characterization shows that the presence of hygroscopic Al_4_C_3_ can be avoided by adding Ti to the composite matrix. The wear behavior of the LMD samples and their microhardness have been compared with Powder Metallurgy samples with the same composition. The LMD samples showed higher hardness and wear resistance.

## 1. Introduction

Additive Manufacturing (AM) is receiving much attention because it could be a way to reduce fabrication costs while reducing raw material and energy consumption, and it could be a solution for fabricating components with difficult geometries [1,2,3,4]. Most research has been carried out in the AM of metallic components; however, in some industrial sectors like the aeronautic and automotive ones, higher performance is required and the use of metal matrix composites (MMC) is an interesting strategy to get higher specific properties to reduce the components weight [5].

However, the use of light alloys in AM has limitations; many aluminum alloys require heat treatments and others are not compatible with AM processes [6]. In castings, the addition of reinforcements to the alloys improves their mechanical properties and increases their stiffness to values that overcome those of heat treated wrought alloys [7,8,9]. Therefore, it is necessary to increase the research about the AM fabrication of MMC components in order to combine the benefits of MMC with those of AM for obtaining net-shape components [10,11,12,13,14].

Aluminum matrix composites reinforced with SiC particles (Al/SiCp) have high tribological properties [15,16,17,18,19,20,21,22]. However, it is known that from 667 to 1347 °C, Al and SiC react and form Al_4_C_3_ and Si [23,24,25]. Al_4_C_3_ is a brittle and hygroscopic intermetallic phase, so its formation must be avoided [26]. Ti has a greater tendency towards the formation of carbides than aluminum, so Ti could avoid the formation of Al_4_C_3_ [27,28,29]. This addition makes that the new phases formed are TiC and TiSi_2_, which are hard and do not degrade in humid ambient, so they improve the final bulk properties, at a difference of Al_4_C_3,_ which is formed when no Ti is added to the powder used.

The aim of this work is the optimization of the processing parameters for the AM of Al/SiCp composite materials. In addition, the goal is to analyze the microstructure and mechanical properties of the samples fabricated and to compare them with powder metallurgy samples fabricated by uniaxial pressing and sintering.

## 2. Materials and Methods 

Hollow cylindrical samples (20 mm in diameter and 20 mm in height) were made by Laser Metal Deposition (LMD) using Al 12 wt.% Si powder with D50 of 71 µm supplied by Metco (52C-NS) (Pfäffikon, Switzerland), SiC with D50 of 26.2 µm supplied by Navarro S.A (F-360) (Madrid, Spain), and Ti of 74 µm supplied by Alfa Aesar (Haverhill, MA, USA). Prior to deposition, powders were previously combined in the proportions shown in Table 1 and mixed in a ball mill for 5 h. 

Al 12 wt.% Si–30wt.% SiC composites (Al/SiCp) and Al 12 wt.% Si 20% Ti–30 wt.% SiC composites (Al-Ti/SiCp) were prepared by LMD using a 1300 W and 940 nm continuous wave diode laser (ROFIN DL013S) (Santa Clara, CA, USA) connected to an ABB IRB2400 robot (Asea Brown Boveri, Zurich, Switzerland). Powders were sprayed coaxially with the laser beam through a coaxial nozzle, Fraunhofer IWS COAX 8 (Fraunhofer Institute for Material and Beam Technology, Winterbergstraße, Dresden, Germany). Argon at 4.5 atm pressure and 0.05 L·s^−1^ flow ratio was used as a carrying gas. Additive manufacturing was made on an AISI 316L build plate (Fundiciones Gómez, La Rioja, Spain) of 10 × 15 × 0.5 (in cm) connected to a hot-plate with a temperature control system (200 °C). The processing parameters analyzed were laser power (600–1000 W) and laser scanning speed (20–50 mm/s).

Table 2 shows the set of fabrication conditions that have been used. All the experiments were repeated three times to evaluate the repeatability and the included one is the most statistically relevant.

The microstructures of the samples were examined by an optical microscope (OM) from Leica DMR (Buffalo Grove, IL, USA), scanning electron microscopy (SEM) from Hitachi S3400N (Tokyo, Japan) equipped with an Energy Dispersive X-ray Spectrometer (EDS) from Brucker AXS X flash Detector 5010 (Billerica, MA, USA), and X-ray diffraction (XRD). The effect of adding Ti to the composite matrix to avoid the matrix-reinforcement reactivity was evaluated by analyzing the microstructure of an Al-Ti/SiCp LMD sample.

Microhardness and wear behavior of the Al-Ti/SiCp sample were analyzed. These properties were compared with those of a sample that had the same composition, but that was manufactured by uniaxial pressing (10 Ton/m^2^) and that was sintered at 500 °C for 2 h (PM sample). Microhardness tests were carried out in a Shimadzu microhardness tester (Kyoto, Japan) by applying 1 gf load for 15 s, using a Vickers prims indenter [30]. Wear tests were carried out on the top of the cylinders under dry sliding conditions on a pin-on-disc Microtest tribometer (Microtest S.A., Madrid, Spain) using a 4 mm diameter steel ball as pin, a load of 10 N, 200 rpm, a wear track with a 5 mm diameter and 200 m of sliding distance. Mass loss was measured by a Sartorius BP 211S scale (Gotinga, Germany) with a precision of ±0.0001 g. The tribometer provided friction coefficients data and the Archard coefficients (*K*) were calculated using Equation (1):

(*V/L*) = *K* (*w/H*) (1)

being: *V*—volume loss; *L*—sliding distance; *w*—applied load; *H*—Vickers microhardness. 

## 3. Results and Discussion

**Figure 1:**
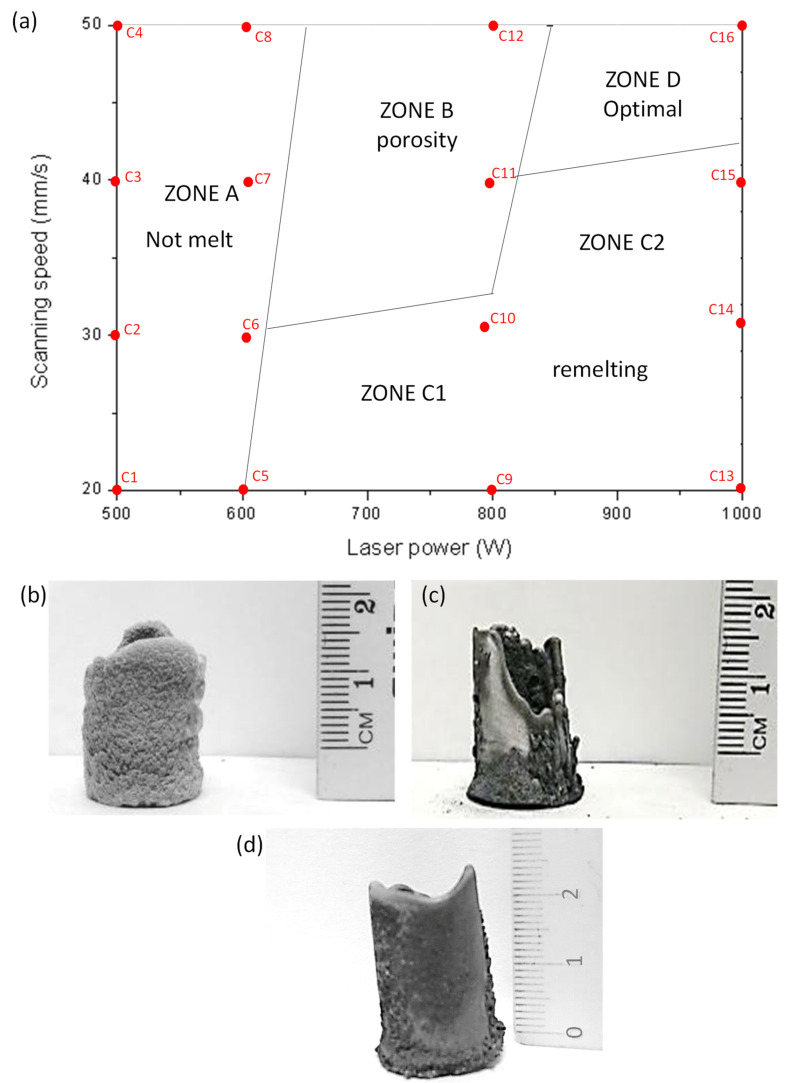
(**a**) Al/SiCp LMD process map; (**b**) sample morphology obtained in zone B; (**c**) sample morphology obtained in zone D; (**d**) sample morphology obtained in zone E.

Figure 1a shows the processing map for the AM of a cylinder sample of 20 mm in diameter and 20 mm in height made of Al/SiCp. The points indicated in the map correspond with the different conditions tested (Table 2). For each condition, the characteristics of the manufactured pieces were analyzed. Figure 1b–d show the main AM behaviors observed in the samples. The building characteristics of the conditions allowed determining the different processing fields observed, and the limits between the zones are approximately drawn.

Laser power and scanning speed directly affect to the building of the manufactured sample because they contribute to the energy density provided to the powder. Locally, also the laser spot size affects the temperature that the powder reaches. The magnitude that comprises all these parameters is the energy density, which is shown in Equation (2).

(2) $$\mathrm{Energy}\;\mathrm{Density}\;(J/{\mathrm{mm}}^{2})=\frac{\mathrm{laser}\;\mathrm{power}\;\left(W\right)}{\mathrm{scanning}\;\mathrm{speed}\;\left(\frac{\mathrm{mm}}{s}\right)\cdot \mathrm{laser}\;\mathrm{spot}\;\mathrm{size}\;\left({\mathrm{mm}}^{2}\right)}$$

At low laser power (<600 W) the sprayed material does not melt regardless of the scanning speed used because the energy density is very low in all cases. The powders were only partially molten and the porosity of the samples was high. The size of the pores observed was similar to that of the powder particles used. Under these conditions manufacturing is not possible (Figure 1a, zone A). 

Between 600 W and 800 W the samples were very porous (Figure 1b) because the sprayed material was only partially melted (Figure 1a, zone B). Using this combination of parameters, the sprayed powders were only partially molten. This had two different effects: the wetting of the SiCp was not achieved and the liquid particles could not deform to fill in the gaps in the microstructure. For this reason, pores with dimensions similar to those of particulates were observed. 

At low scanning speed (<30 mm/s) (Figure 1a, zone C1), with the exception of the lowest energy used, the energy density is high for all the other energies used and the particles and the layers previously deposited are melted. When laser speed and laser power are increased, both compensate as the energy density is maintained constant. Due to this, between 900–1000 W and 30–40 mm/s (Figure 1a, zone C2) the observed behavior was similar to that of zone C1. In both cases (C1 and C2) the energy density is very high, and it exceeds the heat dissipation ability of the AM piece. Therefore, the whole temperature rises, and some layers deposited are re-melted. This occurs in each layer deposited, so it results in the melting of all the piece and material flows by gravity (Figure 1c).

The laser parameters that provide the adequate AM are power in the range 900–1000 W and speeds of 40–50 mm/s (Figure 1a, zone E). Within these conditions, the temperature of the powder is high as the laser power is also high, so it is molten. Furthermore, the laser speed is high, so the input energy is lower than in previous conditions. For these conditions there is an optimal melting of the last deposited layer and a negligible re-melting of the previous ones (Figure 1d). This results in the constant manufacture of the sample with little deformation.

The average energy density can be also controlled by using other strategies, such as pausing the build, modulating the laser energy, or controlling the laser speed. These strategies would have also allowed the cooling of the structure and, possibly the AM of a cylinder, but the microstructure, defects and residual strains would have not been homogenous in it.

**Figure 2:**
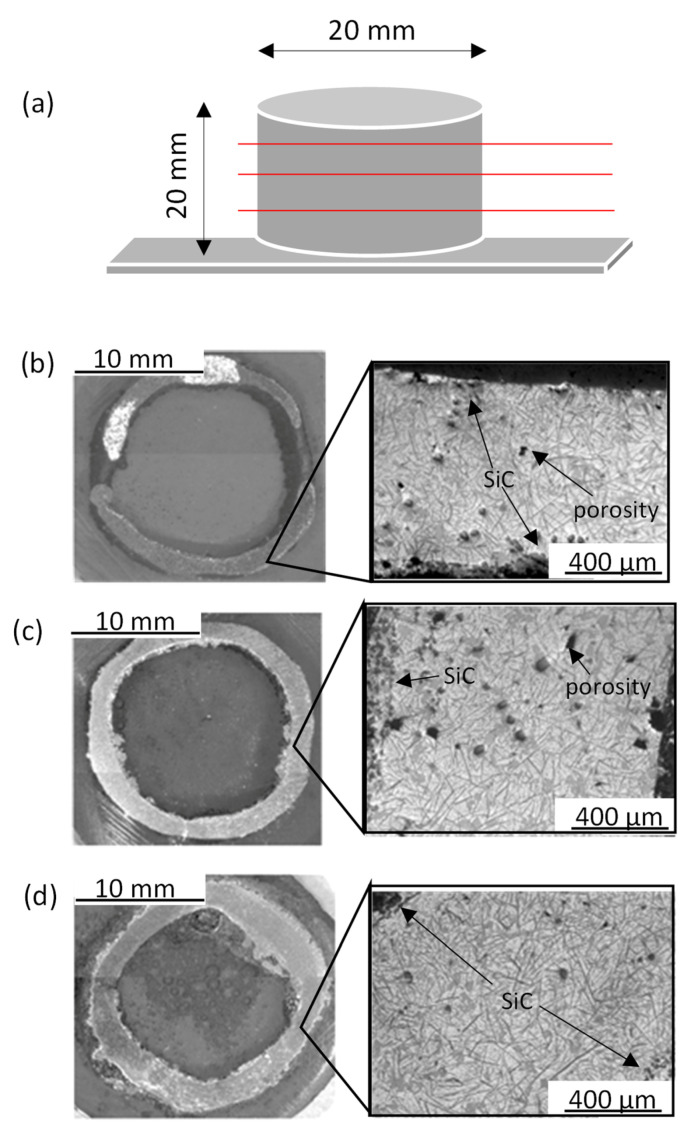
(**a**) Scheme of the cylinder morphology; (**b**) top of the sample cross-section; (**c**) middle of the sample cross-section; (**d**) bottom of the sample cross-section.

Figure 2a shows the scheme of the cylinder morphology. Cross-sections obtained at different points are shown in Figure 2b–d. The cylinder wall thickness decreased from the bottom (near the build plate) to the top, due to higher re-melting in the bottom of the sample. The heat dissipation through the substrate is not favored because stainless-steel has a low thermal conductivity. This makes that the first layers are submitted to high temperatures for longer than the top ones due to the heat transfer of the subsequent layers. For these reasons, these layers are re-melted and their geometry changes during the process. On the other hand, the porosity is lower at the bottom (0.05% ± 0.001%) than at the top (0.5% ± 0.02%). In addition, pore diameter varies with height: pores are smaller at the bottom (−5 µm of diameter) than at the top (−50 µm) due to the re-melting of the bottom layers. 

**Figure 3:**
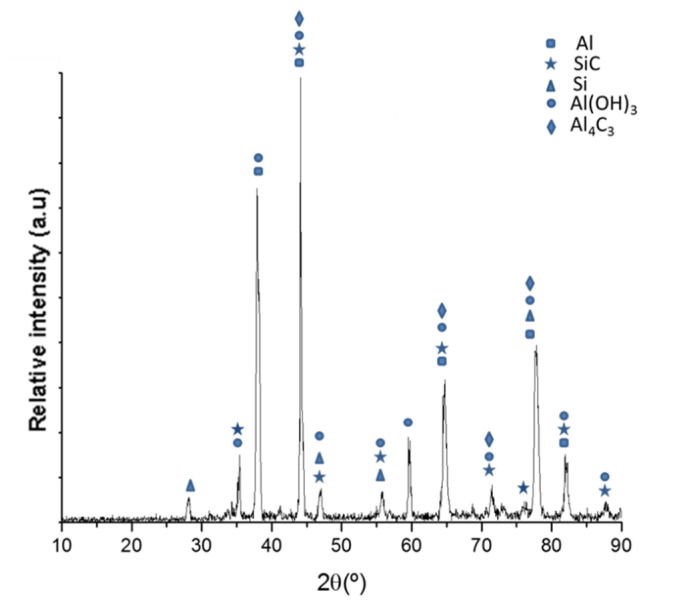
Al/SiCp X-ray diffraction pattern.

Higher laser powers increase the temperature of the molten metal and can cause keyhole collapse, which induces a porosity phenomenon. The microstructure of the material is the same in all the samples, and it does not change with height or with the position in the cylinder wall. The microstructure of the Al/SiCp cylinders was constituted by hypereutectic Al-Si matrix, SiC particles, Si particles, and Al_4_C_3_ (Figure 3 shows the Al/SiCp X-ray diffraction pattern). This indicates that the temperature achieved was above 600 °C [31,32,33]. 

In the inner surface of the cylinder wall, SiC particles totally disappeared as a result of their reaction with the molten Al. At the outside of the cylinder wall there were more SiC particles and less Al_4_C_3_ than at the inside. It is known that the melting-solidification processes are very fast for laser processing [34], but the geometry used makes differences in the inside or the outside of the cylinder wall. The inner cylinder wall can only cool down by conduction heat transport. However, the outer surface can release heat by conduction, radiation, and convection. Therefore, the time that the particles remain above the reaction temperature is less in the outer zone than in the inner one, because the outer ones cool faster. 

**Figure 4:**
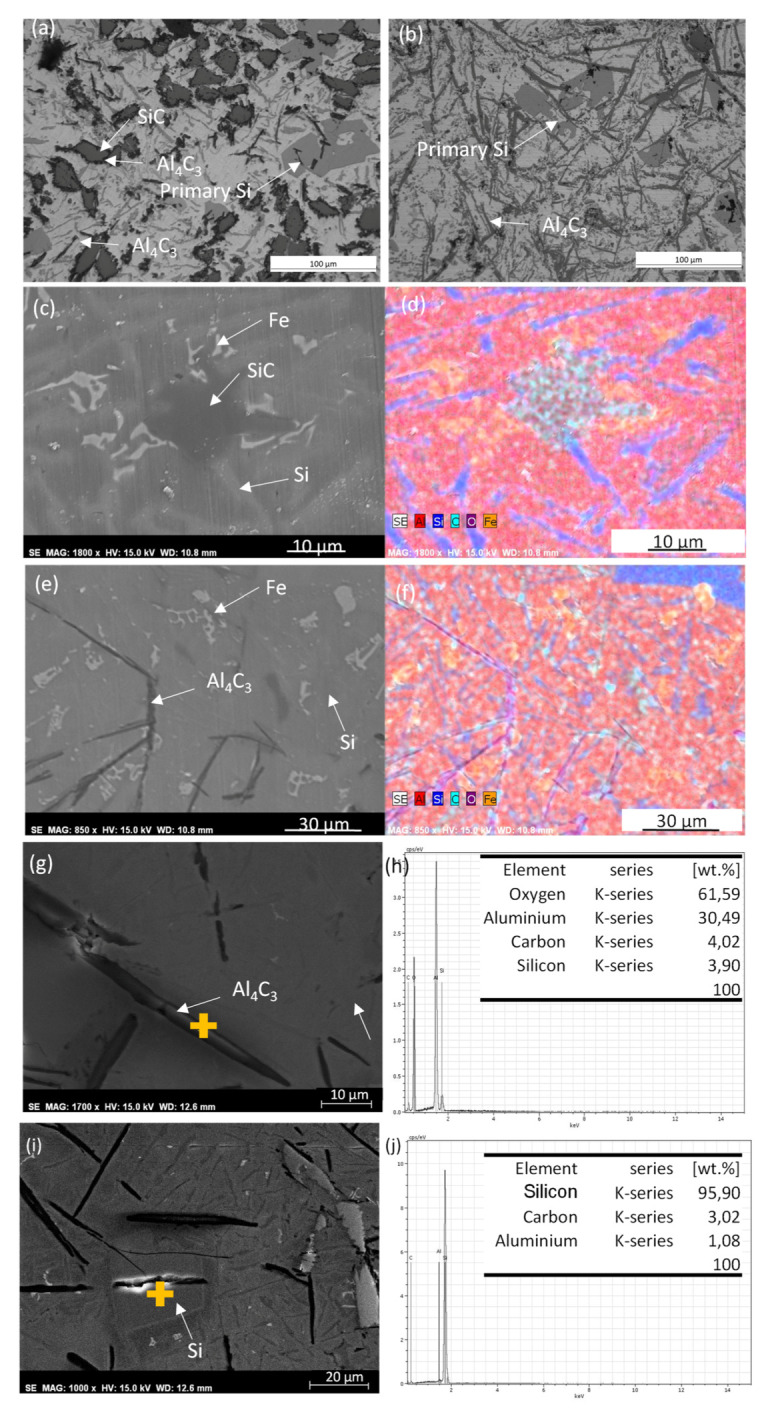
Al/SiCp microstructure (**a**) OM image made on the middle of the cross-section; (**b**) OM image made on the bottom of the cross-section; (**c**) Detail of partially dissolved SiC; (**d**) EDS element map of surface shown in (**c**) color code: aluminum in red, silicon in blue, carbon in cyan, oxygen in purple, and iron in yellow; (**e**) Detail of Al_4_C_3_ needles; (**f**) EDS element map of surface shown in (**e**) color code: aluminum in red, silicon in blue, carbon in cyan, oxygen in purple, and iron in yellow; (**g**) Detail of Al_4_C_3_ needles; (**h**) EDS analysis made on the surface shown in (**g**); (**i**) Detail of primary Si; (**j**) EDS analysis made on the surface shown in (**i**).

Figure 4a shows a detail of the Al/SiCp microstructure, the image was made in the middle of the cross-section. Al_4_C_3_ can be observed around the partially dissolved SiC particles. In addition, little Al_4_C_3_ needles and primary Si can be observed. Figure 4b shows an image of the Al/SiCp microstructure at the bottom of the cross-section in which bigger Al_4_C_3_ needles and primary Si particles can be observed. Figure 4c,d shows a detail of Figure 4a and the EDS elements map made on this surface. A partially dissolved SiCp and eutectic Si can be observed. Figure 4e shows a detail of Figure 4b and the EDS elements map is shown in Figure 4f. Al_4_C_3_ needles and primary Si can be observed. Furthermore, along with the first material layer deposited, the laser melts a little portion of the build plate (weld pool dilution), and for this reason, Fe-rich intermetallic particles were observed at the bottom layers of the sample. The EDS analysis made on the Al_4_C_3_ needles (Figure 4g,h) shows high O_2_ wt.%. This indicates that the Al_4_C_3_ needles are hydrated and, therefore, they are starting to degrade. EDS analysis (Figure 4i,j) showed the presence primary Si, which has been formed due to the reaction between Al and SiC particles.

**Figure 5:**
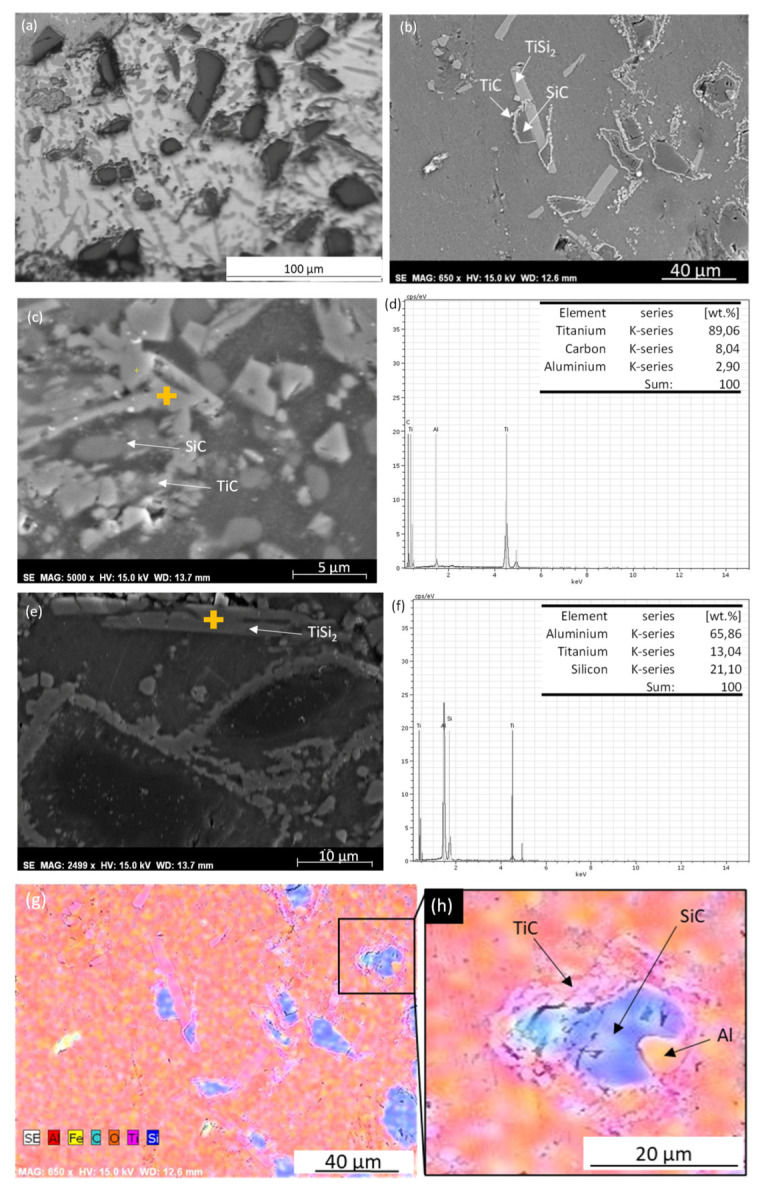
Al-Ti/SiCp (**a**) OM image, microstructure general view; (**b**) SE image; (**c**) detail of TiC particles; (**d**) EDS analysis made on the surface shown in (**c**); (**e**) Detail of TiSi_2_ particles; (**f**) EDS analysis made on the surface shown in (**e**); (**g**) EDS elements map; color code: aluminum in red, silicon in blue, carbon in cyan, oxygen in orange, titanium in purple, and iron in yellow; (**h**) detail of SiC particle.

Figure 5a shows a general view of the Al-Ti/SiCp microstructure and Figure 5b shows a detail of the surface. In them, the Al-Si eutectic matrix can be seen. Furthermore, partially dissolved SiC particles surrounded by S TiC and TiSi_2_ can be also observed, but Al_4_C_3_ was not formed. Similar microstructures (same phases and forms) have been observed in previous research [35,36,37]. During manufacturing, Al reacts with SiC and forms Al_4_C_3_ and Si. Ti reacts with SiC and forms TiC. TiC is more stable than Al_4_C_3_; so, Ti reacts with Al_4_C_3_ to form TiC and Al. For this reason, around the SiCp there was an inner ring rich in Al and an outer ring in TiC (Figure 5c). Figure 5d shows the EDS analysis made on this particle. In addition, Ti reacts with Si and forms TiSi_2_ (Figure 5e). Figure 5f shows the EDS analysis made on this particle. Figure 5g,h shows an EDS elements map of the Al-Ti/SiCp microstructure were the different phases can be observed. Furthermore, as in the case of the Al/SiCp sample, Fe-rich intermetallic phases have been observed in the first deposited layers.

**Figure 6:**
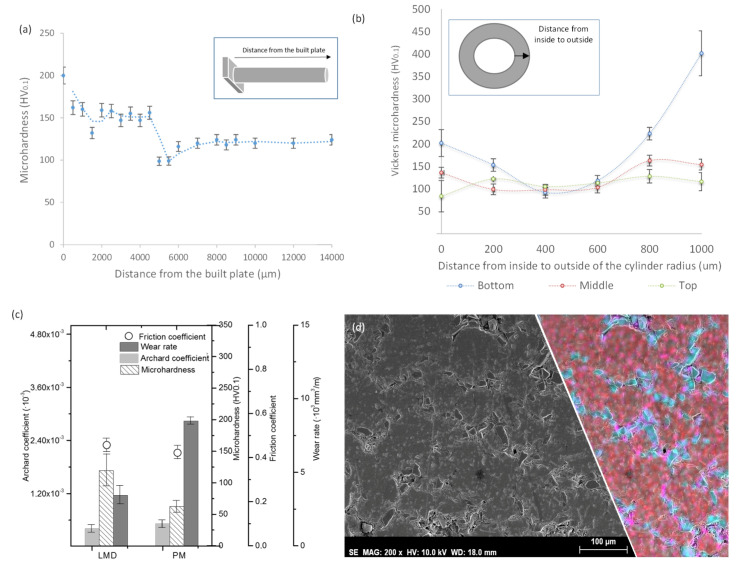
(**a**) Microhardness evolution across the cylinder longitudinal direction; (**b**) Microhardness evolution from inside to outside of the cylinder radius; (**c**) wear test measurements: wear rate (dark grey columns), Archard coefficients (light grey columns), microhardness (lined columns) and friction coefficients (circles) for PM and LMD samples; (**d**) PM sample microstructure, SE image and EDS elements map, color code: aluminum in red, silicon in cyan and, carbon in magenta.

Figure 6a shows that the Al-Ti/SiCp microhardness decreases along the cylinder from the bottom to the top. The first 400 mm are influenced by the presence of Fe rich intermetallic precipitates from the substrate due to the Marangoni flow. This effect is a convective mechanism produced under the laser beam where the temperature of the molten pool is at its highest level and the surface tension is at its lowest value. The temperature of the liquid decreases from the center to the edge of the molten pool and increases the surface tension. This produces that the liquid pulls away from the center of the beam and flows around the molten pool. The microhardness increases from the first 400 mm to the top of the sample due to the decrease in the grain size in the microstructure, due to the higher cooling rate.

Figure 6b shows the microhardness evolution across the cylinder wall (from the inside to the outside). The three lines shown in Figure 6b are correlated with the three cross-sections shown in Figure 2b–d. In the cross-section at the bottom, the microhardness is higher at both sides of the cylinder wall and is lower at center. This behavior is correlated with the microstructure observed in the cross-section. Either Al_4_C_3_ needles observed in the inner wall and SiC particles accumulated in the outer wall cylinder increase the hardness of the material. However, the former one results in the degradation of the material, while the latter one is more stable. These effects make that the outer zones of the cylinder wall are harder than its center.

The same behavior was observed in the middle cross-section; however, in this zone, heat accumulation was lower and there were more and better dispersed SiCp. In average, hardness in this zone was lower. Finally, the top cross section showed a constant microhardness because of the lower heat accumulation and the better SiCp distribution.

Figure 6c shows the results obtained from the wear tests. The wear rates (dark grey columns), Archard coefficients (light grey columns), microhardness (lined columns), and friction coefficients (circles) were analysed. LMD samples were compared with a sample with similar composition but fabricated by uniaxial pressing and sintering. The Al-Ti/SiCp LMD sample is harder and the wear rate is lower than the PM sample (Figure 6c). The friction coefficient was similar in both samples, but the Archard coefficient was lower in the LMD sample, suggesting that the wear mechanism was softer. The PM sample microstructure shown in Figure 6d suggests that the reason for this wear behaviour is the better particle union due to the melting during LMD fabrication and also the low sintering obtained during the PM fabrication.

**Figure 7:**
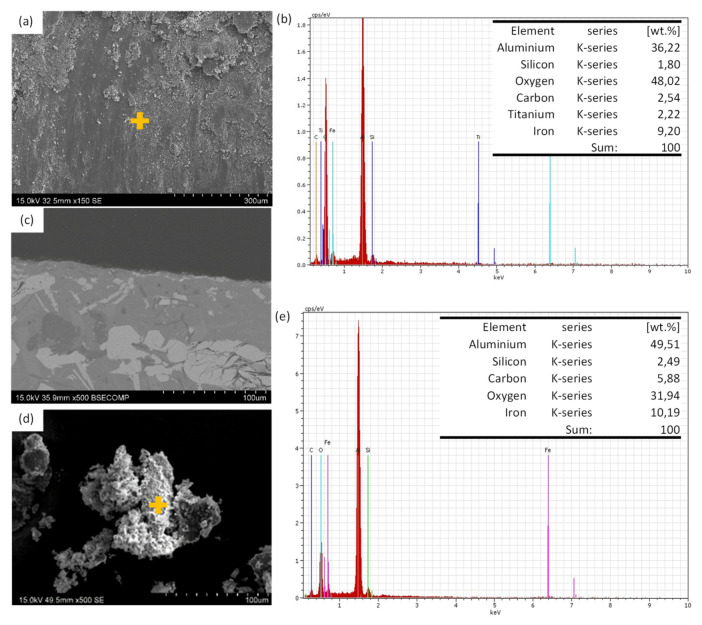
SEM micrographs of wear surfaces of LMD sample (**a**) wear surface; (**b**) EDS analysis made on surface shown in (**a**); (**c**) wear mark cross section; (**d**) wear debris; (**e**) EDS analysis made on debris shown in (**d**).

Figure 7a shows the wear track formed on the surface of the LMD sample. The wear mechanism was abrasive and oxidative (oxygen is observed in the EDS analysis, inset Figure 7b). A thin oxidation layer was observed in the cross-section shown in Figure 7c. In addition, in some areas, delamination mechanism was observed (Figure 7a). The LMD sample did not showed delamination due to its plastic deformation capacity and due to the good integration of the reinforcement in the metal matrix, as it is shown by the wear rate data and Archard coefficient (Figure 6c). In addition, debris was flake shaped and its surface was oxidized, while EDS showed presence of Fe from the pin (Figure 7d,e). 

**Figure 8:**
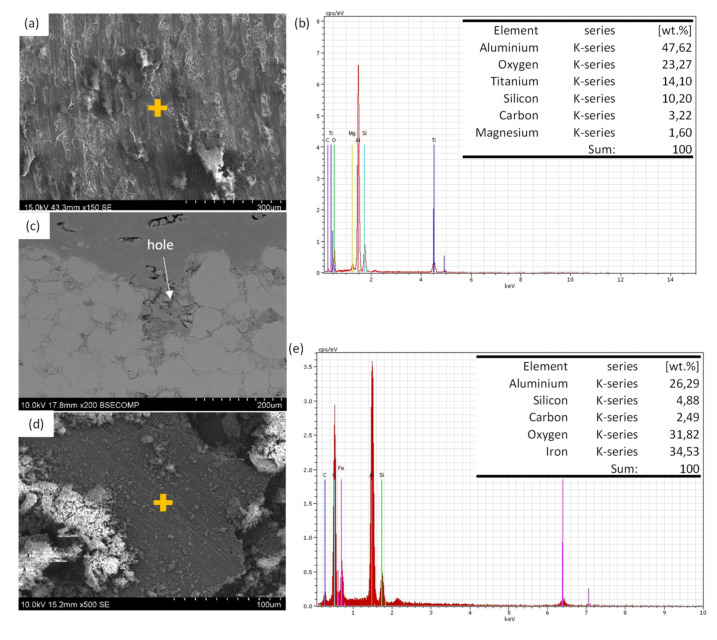
SEM micrographs of wear surfaces of PM sample (**a**) wear surface; (**b**) EDS analysis made on surface shown in (**a**); (**c**) wear mark cross section; (**d**) wear debris; (**e**) EDS analysis made on debris shown in (**d**).

Figure 8a shows the wear surface on PM sample. Like in the other case, the wear mechanism was abrasive and oxidative but oxygen percentage is lower than in LMD sample as is shown in Figure 8b). In addition, in some areas, a delamination mechanism has been observed with much more relevance than in the LMD samples. In the PM ones, SiCp was pulled out and big cracks were formed (Figure 8c). Similar results have been obtained in Al_2_O_3_ with Ni and Cu inclusions coatings [38] were a decrease of hardness and wear resistance in porosity samples have been observed. In addition, like in the LMD sample, the debris was flake shaped and its surface was oxidized. Furthermore, the EDS made on the debris showed presence of Fe (Figure 7d,e).

## 4. Conclusions

In summary, a processing map for Al/SiC_p_ samples fabricated by Direct Laser Deposition that relates the laser power and the scan speed has been developed. The cylindrical shape was chosen as it represents a general structure. Laser powers higher than 600 W and scanning speed below 30 mm/s, result in re-melting samples. Laser powers lower than 600 W result in insufficient fusion and very porous samples were obtained as they were not molten. Powers between 600 and 800 W and scanning speeds higher than 30 mm/s resulted in samples with high porosity. The homogeneous growth of the piece appears when two main features are simultaneously achieved: the laser power is high enough to heat the powder above its melting temperature and to allow the metal to flow and close defects; and the energy density is low enough to avoid the re-melting of the previously manufactured layers. This fabrication zone is restricted to laser powers between 800 to 1000 W and scanning speeds between 40 and 50 mm/s. 

The microstructure of the samples is determined by the heat-cooling process. Cooling is faster at the outside face of the cylinder wall, so there are more SiC particles and less Al_4_C_3_ than in the inside face. The presence of partially dissolved SiC particles and Al_4_C_3_ increase the microhardness. No detrimental Al_4_C_3_ was formed in the Al-Ti/SiCp sample.

The AM cylinder was harder, and its wear behaviour was better than PM ones with a identical composition. The friction coefficient was similar in both samples; however, the Archard coefficient was lower in the LMD sample, showing that its wear mechanism is softer.

## Figures and Tables

**Table 1:** Projected powder proportions.

Percentage	Abbreviated Name
Al 12 wt.% Si–30 wt.% SiC	Al/SiCp
Al 12 wt.% Si–20 wt.% Ti–30 wt.% SiC	Al–Ti/SiCp

**Table 2:** Set of experiments.

Condition	Laser Power (W)	Scanning Speed (mm/s)
C1	500	20
C2	500	30
C3	500	40
C4	500	50
C5	600	20
C6	600	30
C7	600	40
C8	600	50
C9	800	20
C10	800	30
C11	800	40
C12	800	50
C13	1000	20
C14	1000	30
C15	1000	40
C16	1000	50
